# *Enterobacter cloacae* infection of the shoulder in a 52-year-old woman without apparent predisposing risk factor: a case report and literature review

**DOI:** 10.1186/s12879-020-05699-9

**Published:** 2021-01-06

**Authors:** Jingjie Huang, Qiliang Xu, Fuyifei Liu, Hao Xiong, Junxing Yang

**Affiliations:** 1 The Sixth Clinical Medical College, Guangzhou University of Chinese Medicine, Shenzhen, China; 2 Department of Orthopedics, Shenzhen Hospital of Guangzhou University of Chinese Medicine (Futian), Shenzhen, China; 3 Department of Orthopedics, The First Affiliated Hospital of Guangzhou University of Chinese Medicine, Guangzhou, China

**Keywords:** *Enterobacter cloacae*, Septic arthritis, Shoulder

## Abstract

**Background:**

*Enterobacter cloacae* (*E. cloacae*) is one of the commensal flora in the human intestinal tract and a prevalent nosocomial pathogen, which rarely causes infectious osteoarthritis in immunocompetent patients without recent trauma or surgery. Here, we report the first case of septic monoarthritis of the shoulder caused by *E. cloacae* in an immunocompetent patient.

**Case presentation:**

A 52-year-old female with a 6-year history of right shoulder pain was referred to our emergency department due to fever, acute severe shoulder pain, and swelling. Blood test showed elevated inflammatory markers. The patient denied any recent invasive surgical procedure and trauma. She was misdiagnosed with a frozen shoulder, and the anti-inflammatory painkiller celecoxib for symptomatic treatment was ineffective. Magnetic resonance imaging (MRI) showed a shoulder joint abscess and supraspinatus tendon tear. The joint aspirate culture showed *E. cloacae*. After late diagnosis, she was treated with levofloxacin and underwent surgical debridement and irrigation. Her follow-up data revealed that she did not suffer from shoulder swelling and severe pain.

**Conclusion:**

This is a rare case of *E. cloacae* infected arthritis of the shoulder in an immunocompetent patient with a rotator cuff tear, indicating that even if the symptoms and age of the patients match the characteristics of frozen shoulder, the possibility of septic arthritis should be considered in the presence of fever and increasing inflammatory markers. The cases of our literature review suggest that the patients subjected to invasive procedure may develop a subsequent *E. cloacae* osteoarticular infection, regardless of being asymptomatic after the procedure.

## Background

*E. cloacae* is an anaerobic Gram-negative bacterial strain primarily found in the intestinale tract and widely distributed in environments such as soil, water, and sewage [1]. Reports have found that the bacteria contaminate various medical devices, thereby causing nosocomial outbreaks due to the colonization of certain operative cleaning solutions and surgical equipment [2–6]. Over the past decades, it has emerged as one of the most common nosocomial pathogens, infecting patients with underlying diseases, immunosuppression, and prolonged hospital stay especially in ICU and burns ward [1, 2]. The bacteria generally cause sepsis, urethritis, and lower respiratory tract infection, but rarely causes septic osteoarthritis [1]. However, accumulating evidence shows that it is also a common source of infection in orthopedic departments [3, 7–10].

Frozen shoulder is a condition with pain and limited movement, which is relatively prevalent in women aged between 40 and 65 [11]. These clinical characteristics are similar to those of rotator cuff tear. The patient in this study is a 52-year-old female with a 6-year history of right shoulder pain and limited movement. Before MRI identified an abscess and rotator cuff tear, she was diagnosed with a frozen shoulder and received an ineffective treatment.

To our knowledge, this is the first documented case that *E. cloacae* arthritis of the shoulder in an immunocompetent patient, and she had no apparent predisposing risk factors.

## Case presentation

**Fig. 1:**
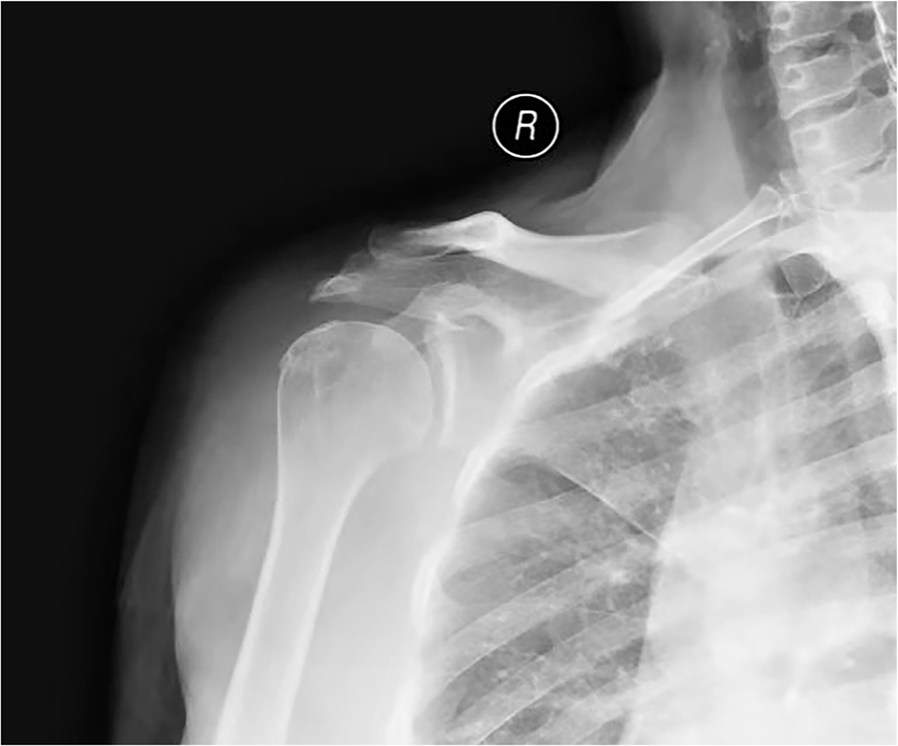
Posteroanterior view showing degenerative change

A 52-year-old female was admitted to our emergency department with fever, sore throat, acute severe right shoulder pain with a burning sensation, redness and swelling. She had a medical history of hypertension, hyperlipemia, renal lithiasis, and hydronephrosis, for which she was daily prescribed with levamlodipine besylate and atorvastatin calcium. She was not found with any common risk factors of septic arthritis such as immunosuppression, diabetes, recent trauma or surgery. Six years ago, she felt chronic pain at the right shoulder and limited movement without inducement. About a month before the acute episode, she was administered with right deltoid muscle injections of triamcinolone acetonide in a local clinic five times to relieve the recently worsened pain, after which the symptoms relieved temporarily while recurrent fevers occurred. Two days after the last dose, she experienced severe pain on her shoulder joint with minimal passive movement, local skin temperature increased with redness, and had a sore throat. X-ray films of the right shoulder joint were examined (Fig. 1), where degenerative change of the right shoulder joint was observed. Her body temperature was 38.3 °C, the blood test showed increased level of WBC at 12.70 × 10^9^/L (normal range 4.0–10.0 × 10^9^cells/L, and Erythrocyte Sedimentation Rate (ESR) of 65 mm/h (normal range 0–20 mm/h) and C-Reactive Protein (CRP) level substantially increased to 41.2 mg/dL (normal range < 0.8-8 mg/dL). Based on the blood test results and her symptoms, she was diagnosed with a frozen shoulder associated with upper respiratory tract infection. After taking the anti-inflammatory painkiller Celecoxib for 8 days, her condition had no improvement.

**Fig. 2:**
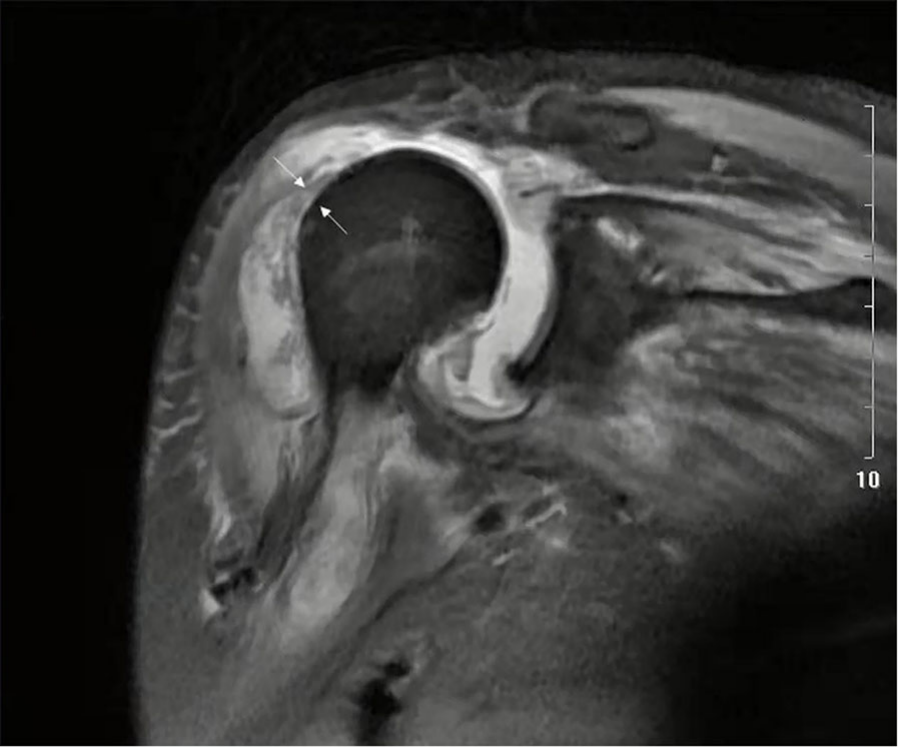
MRI of the right shoulder showing supraspinatus tendon tear (arrow) and massive abscess

For systemic treatment, she was referred to the orthopedic department. MRI results showed a shoulder joint abscess (Fig. 2). A total of 26 mL (normal range 0.1-2 mL) red gross pus was drawn from her joint for analysis. In total,46,000 cells/mm^3^ (normal range 200–700 cells/mm^3^) WBCs, 97% (normal range < 25%) polymorphonuclear (PMN) leukocytes and *E. cloacae* on culture were identified, whereas the fungal culture test was negative. The results of the antibiotic sensitivity test of the cultured pathogen are shown in Table 1. Based on these physical, clinical, radiological findings, and laboratory tests, a diagnosis of septic arthritis was made, and a surgical treatment plan, including arthroscopic debridement and irrigation, was administered. Through the arthroscopy, a synovial proliferation of glenohumeral joint, sporadic faint yellow floccule, and a massive rotator cuff tear was observed. In consideration of the shoulder joint infection, rotator cuff tear repair was not performed immediately, nevertheless, the intra-articular space was sufficiently irrigated. Based on the result of the antimicrobial susceptibility test (Table 1), intravenous injection of levofloxacin (300 mg, q12h) was administered to the patient from the day of surgery. After 5 days, her shoulder pain was significantly relieved and her body temperature normalized. After discharge, she orally took levofloxacin antibiotics (0.5 g, qd).

**Table 1:** Antimicrobial Susceptibility Testing of *E. cloacae* cultured from joint aspiration

Antimicrobic	MIC(μg/ml) ^a^	Kirby-Bauer method (mm)	Interpretation ^b^
Cefazolin	≥64		R
Ceftazidime	≤1		S
Cefepime	≤1		S
Aztreonam	≤1		S
Piperacillin/Tazobactam	≤4		S
Cefoperazone/Sulbactam		30	S
Imipenem	≤1		S
Ciprofloxacin	≤0.25		S
Levofloxacin	≤0.25		S
Gentamicin	≤1		S
Tobramycin	≤1		S
Amikacin	≤2		S
Nitrofurantoin	32		S
Ceftriaxone	≤1		S
Ertapenem	≤0.5		S
Cefotetan	≤4		R
Trimethoprim/Sulfonamides	≤20		S

^a^Minimal Inhibitory Concentrations, b Interpretation was according to CLSI breakpoints (CLSI M100S-25)

Unfortunately, the symptoms including increasing pain, joint swelling, and increased local skin temperature recurred after 12 days. Her body temperature increased to 38.1 °C, the WBC level was 11.72 × 10^9^/L, ESR increased to 75 mm/h, and the CRP level rose to 69.1 mg/L. As a result, 6.5 mL red gross pus was drawn from the joint which showed 60,764 cells/mm^3^ WBCs with 96% PMN leukocytes and negative culture. Given that the wound left by her last surgery had not entirely healed, an intravenous injection of Levofloxacin (300 mg, q12h) was again administered instead of the surgical debridement and irrigation. Her condition gradually improved during re-hospitalization. After 19 days, her symptoms significantly relieved, i.e., the WBC level decreased to 8.66 × 10^9^/L, ESR decreased to 19 mm/h, and CRP decreased to 3.53 mg/L. As a consequence, the intravenous injection was changed to oral administration. On the 24th day of re-hospitalization, she was discharged and orally prescribed with levofloxacin (0.5 g, qd) for 2 weeks. After discharge, the patient was followed-up by telephone for2 years. Although she refused to undergo the operation of rotator cuff tear repair, follow-up data revealed that she did not suffer from shoulder swelling and severe pain anymore, however, the mild pain and movement restriction persisted.

## Discussion and conclusion

*E. cloacae* is an opportunistic pathogen causes various nosocomial infections, and has been considered as a rare causative of infections in the orthopedic unit. However, emerging evidence shows that *E. cloacae* is also a frequent causative agent of osteoarthritis. Notably, *E. cloacae* is the third most common (9.5%) organism of intraoperative bone culture from chronic osteomyelitis patients in a south China hospital [7]. In a different Chinese hospital, *E. cloacae* accounted for up to 10.6% of post-traumatic osteomyelitis cases [8]. Also, a retrospective case series showed that *E. cloacae* was the most prevalent source of healed fracture infection after internal fixation (29.4%, 5/17) [9]. The *E. cloacae* osteoarticular infections are mainly caused by direct inoculation attributed to invasive procedures such as trauma and surgery.

Septic arthritis of the shoulder is relatively rare [2]. In this study, we report the first case of monoarthritis of the shoulder caused by *E. cloacae* in an immunocompetent patient, with no apparent risk factors. To better understand the characteristics of *E. cloacae* infected bones and joints in patients without recent post-traumatic or post-operative medical history, a PubMed search was conducted and a total of 13 cases with detailed information, summarized in Table 2 were identified [12–24]. The literature review indicated that, including our patient, the ratio of male/female among the 14 patients without a pertinent history of open procedure was 5:2. Ten of the patients (No.3–6, No.8, No.9, No.11–14) showed apparent risk factors including multifocal infection, immunosuppression, sepsis, or even organ failure. However, 3 of the reported patients (No.2/7/10) had septic osteoarthritis due to trauma or hematogenous seeding at the same site or nearing site several years ago, and they were asymptomatic until the current episodes. These cases remind us that those have undergone invasive procedure may have a subsequent osteoarticular infection, even if they have been asymptomatic after the procedure.

**Table 2:** Summary of *E. cloacae* osteoarthritis in patients without post-traumatic or post-operative medical history

No.	Gender	Age (years old)	The site(s) of osteoarthritis	Comorbidities	Chief complaints/Symptoms	Sample of microbiological test	Pathogen (s)	Antibiotic treatment	Inflammatory markers on admission	The isolated or cultured *E. cloacae* sensitive antibiotics	Outcome
1 (our case)	F	52	Right shoulder	Had right and left surgical kidney stone removal in 7 and 15 years ago. Rotator cuff tear.	6-year history of right shoulder pain and restricted movement, worsened in the last month. Fever, sore throat. Burning sensation, swelling and redness around the right shoulder	Joint aspiration	E. Cloacae	Levofloxacin (300 mg, IV, q12h, 5 days) → 0.5 g, po, qd, 11 days → 300 mg, IV, q12h, 9 days → 0.5 g, po, qd, 17 days	WBC:12.7 × 10^9^/L, CRP:41.2 mg/dL, ESR:65 mm/h	Details in Table 1	Recovery
2 [12]	M	2	Left knee	Osteoarticular infection at the same site 2 years earlier (no organism was identified), and he was born via forcep delivery	Limp and left knee pain and swelling for 2 weeks	Joint aspiration and synovial tissue	E. Cloacae	Meropenem(20 mg/kg, IV, q8h, 3 weeks) → Sulfamethoxazole–Rimethoprim (4 mg/kg, q12h, po, 6 months)	NA	Meropenem, cefepime, ciprofloxacin and trimethoprim-sulphamethoxazole	Recovery
3 [13]	F	36	Right shoulder	HIV-positive, sickle cell anemia, latent tuberculosis infection	Fever, weight loss, fatigue, 4-months history of right shoulder pain	Joint aspiration and surgical specimens	E. Cloacae and *Mycobacterium tuberculosis*	Imipenem and Amikacin for 2 weeks, IV	CRP:0	Imipenema, latomoxef, amikacin, pefloxacin and ciprofloxacin	Recovery
4 [14]	M	14	Left sacroiliac joint	Sepsis	1-day history of fever and hip pain aggravated by walking, ARDS happened on the third hospital day	Blood	E. Cloacae	Vancomycin(4 g/day) and Ceftriaxone (4 g/day) → Ceftriaxone (4 g/day) and Amikacin (1.5 g/day) for 6 weeks	WBC: 6.4 × 10^9^/L, ESR:12 mm/h, CRP:3.1 mg/dL	Amikacin, aztreonam, ceftriaxone, ceftazidime, cefotaxime, ciprofloxacin, gentamicin, imipenem, piperacillin/tazobactam;	Recovery
5 [15]	M	88	T10/T11	Long-term urinary catheter, malignancy	Back pain, fever, rigors, weight loss	Blood	E. Cloacae	Meropenum (IV, 3 weeks) and Ciprofloxacin (Long time)	WBC: 10.06 × 10^9^/L, CRP: 227 mg/dL	NA	Failed to therapy
6 [16]	M	54	C3-C4	Meningitis and sepsis secondary to urinary tract infection after transrectal ultrasound and biopsies, he had a raised PSA level and acute renal failure	10-day history of headaches, dizziness, neck pain and altered sensation in his upper, limbs	Blood	E. Cloacae and Klebsiella oxytoca	Ciprofloxacin and metronidazole for 5 days → Ceftriaxone (IV, 2 weeks) and a longer course of oral ciprofloxacin	NA	NA	Recovery
7 [17]	M	57	leg	MSSA infection at the same site after an open fracture 31 years ago. After successful treatment, the facture healed and he remained asymptomatic until the present episode. The implant material was removed many years ago	1-week history of leg pain, swelling and local tenderness but no inflammation of the overlying skin or draining fistula	Bone	E. Cloacae	Garamycin (3 weeks) and Cefepime (6 weeks)	NA	NA	Recovery
8 [18]	M	52	L5-S1	Had a extracorporeal shock wave lithotripsy due to right renal lithiasis and hydronephrosis	Chills, shaking, high fever, back pain, restricted lumbar movements	Blood and urine	E. Cloacae	Amikacin (1500 mg/day) Indometacin (150 mg/day) for 1 week → Ceftriaxone (2 g, q12h, 1 week) → 1 g/day,3 months)	WBC:18 × 10^9^/L, CRP:12.3 mg/dL ESR:110 mm/h	Ceftriaxone and amikacin	Recovery
9 [19]	M	47	Multiple joints	Acute pancreatitis, multi-organ failure, ARDS, systemic fatty necrosis	High fever, chills, low tension, tachycardia, painful erythematous nodules on the arms, thighs, ankles and fingers	Blood and joints aspiration	E. Cloacae	Pefloxacin, metronidazole and amoxicillin→imipenem-cilastatin	WBC: 5.2 × 10^9^/L, CRP:201 mg/dL	NA	Dead
10 [20]	M	50	Cervical spine	Hypertension, arthritis, and a gunshot wound to the left chest and birdshot to the head and neck 20 years earlier that resulted in a seizure disorder, and the pellet had not been removed	4-day history of neck pain spreading to right temporal region, right shoulder, right lateral chest, and right upper back from the scapula to the midthoracic spine, mild dysphagia	Blood	E. Cloacae	NA	WBC:7.9 × 10^9^/L	NA	Recovery
11 [21]	M	10 weeks old	Left proximal tibia	Watery stools, malnourished and dehydration 5 weeks earlier. Intraosseous (IO) needle had been placed into the proximal left tibia and a permanent Silastic intravenous catheter had been inserted to start parenteral nutrition and antibiotics	Desquamating dermatitis, erythematous nodules on the back skin, the left lower extremity was erythematous and indurated	Blood, bone and the serosanguinous aspiration expressed by the IO needle	*Candida albicans* and E. cloacae	Ampicillin and cefotaxime(IV) → Ticarcilin-clavulanate(IV) → Fluconazole and aztreonam → amphotericin B and aztreonam	NA	NA	Recovery
12 [22]	M	28	L4-L5	HIV-positive, intravenous heroin abused, hepatitis C	2-month history of severe low back pain, fever, night sweats, and weight loss. The lumbar spine was markedly tender with bilateral paravertebral muscle spasm	Joint aspiration	E. Cloacae	Amikacin (1 g/day, intramuscular, 3 weeks) and pefloxacin (800 mg/day, IV, 3 weeks) → pefloxacin ((800 mg/day, po, 14 weeks)	WBC:9.2 × 10^9^/L, ESR:50 mm/h	Amikacin, pefloxacin, and trimethoprim-sulphamethoxazole	Recovery
13 [23]	F	68	T8-T9	Gallstones with repeated hepatic colic for 3 years. Intravenous urography, and a barium enema had been done	2-week history of severe pain in the right hypochondrium that increased with motion, general malaise, anorexia, and dystermia. Fever after barium enema	Blood and Joint aspiration	E. Cloacae	trimethoprim(160 mg, q12h, IV, 10 days) and sulphamethoxazole (800 mg, q12h, IV, 10 days) → (trimethoprim-sulphamethoxazole, po)	WBC:5.9 × 10^9^/L, ESR:127 mm/h	Gentamicin and trimethoprim-sulphamethoxazole	Recovery
14 [24]	F	Prematre neonate (28 weeks)	Multiple joints	Premature, hyaline membrane disease, sepsis. Umbilical artery catheter was inserted, continuous positive airway pressure using nasal prongs, intravenous nutrition	Septic shock, cyanosis, tachypnea, grunting, erythematous left ankle, knee effusion, lost passive and active motion in hips	Blood, joints aspiration (Hips, right knee, right ankle) and tip of the catheter	E. cloacae (blood, joints aspiration and tip of the catheter) Klebsiella pneumonia (blood)	Gentamicin (2 mg, q8h, IV, 6 days) and Methicillin (IV, 6 days) → Furadantin (2.5 mg, q6h, IV, 9 days) → Furadantin (2.5 mg, q6h, IV) and Nalidixic acid	NA	Furadantin and Nalidixic acid	Recovery

*MSSA* Methicillin-sensitive S. aureus, *PSA* Prostate-specific antigen, *WBC* White blood cell, *CRP* C-reactive protein, *ESR* Erythrocyte sedimentation rate, → From the former treatment changed to the latter, *NA* Not available

The case presented here is complicated, where the patient is a middle-aged woman with chronic pain in the shoulder joint for 6 years, and was routinely diagnosed as a frozen shoulder. Later, the symptoms of fever and sore throat on admission masked the infection of the shoulder, she was misdiagnosed with a frozen shoulder and upper respiratory tract infection. Treatment with steroid and anti-inflammatory painkillers was ineffective. She exhibited no apparent risk factors for shoulder joint infection, thereby increasing the difficulty of diagnosis. Therefore, our case informs orthopedists that the possibility of septic arthritis should be considered in instances where fever and increasing inflammatory markers are detected.

It remains unclear how *E. cloacae* infected the shoulder joint of an immunocompetent woman who denied any recent open surgery or penetrating trauma. Additionally, the patient had a massive rotator cuff tear, suggesting that her pain for 6 years might potentially attributed to the ruptured rotator cuff. She was five times administered with right deltoid muscle injections of triamcinolone acetonide in a local clinic, which subsequently followed by acute arthritis. Anatomically, the massive rotator cuff tear permitted direct communication between the joint cavity and the subdeltoid bursa, which perhaps makes it easier for bacteria to enter the joint cavity from the deltoid muscle region [25]. Corticosteroid injection is a potential risk for osteoarthritis [26]. Moreover, there were cases reporting soft-tissue infections after intramuscular injection [27–29]. But it is worth noting that the patient in this study did not develop a local infection after intramuscular injection. Danilo et al. reported 7 patients with rotator cuff tear developing shoulder joint infection without trauma or surgery of shoulder [30]. Nonetheless, despite the absence of evidence, their report cannot clarify the relationship between rotator cuff tears and infection. The damaged shoulder joints due to rotator cuff tear might however have a higher risk of infection, but additional clinical research is required to support this speculation.

To our knowledge, this is the first documented case of *E. cloacae* monoarthritis of the shoulder in an immunocompetent patient, with no apparent risk factors such as recent shoulder surgery or trauma, diabetes, intravenous substance abuse, malignancy, and immunosuppression. The case reminds us when the subclinical inflammatory markers increase, even if the patient’s clinical features are consistent with the frozen shoulder, the possibility of septic arthritis should be considered. Furthermore, our review suggests that the patients subjected to invasive procedures might develop a subsequent osteoarticular infection, regardless of being asymptomatic after the procedure.

## Data Availability

The main data generated or analyzed in this case report are included in the article. More detailed data are available from the corresponding author on a reasonable request.

## Abbreviations

CLSI: Clinical and laboratory standards institute
CRP: C-reactive protein
ESR: Erythrocyte sedimentation rate
IO: Intraosseous
MRI: Magnetic resonance imaging
PMN: Polymorphonuclear
WBC: White blood cell
